# Carboxyhemoglobin (CO-Hb) Correlates with Hemolysis and Hospital Mortality in Extracorporeal Membrane Oxygenation: A Retrospective Registry

**DOI:** 10.3390/diagnostics12071642

**Published:** 2022-07-05

**Authors:** Xavier Bemtgen, Jonathan Rilinger, Manuel Holst, Felix Rottmann, Corinna N. Lang, Markus Jäckel, Viviane Zotzmann, Christoph Benk, Tobias Wengenmayer, Alexander Supady, Dawid L. Staudacher

**Affiliations:** 1 Interdisciplinary Medical Intensive Care (IMIT), Medical Center—University of Freiburg, Faculty of Medicine, University of Freiburg, 79106 Freiburg, Germany; corinna.nadine.lang@uniklinik-freiburg.de (C.N.L.); viviane.zotzmann@uniklinik-freiburg.de (V.Z.); tobias.wengenmayer@uniklinik-freiburg.de (T.W.); alexander.supady@uniklinik-freiburg.de (A.S.); dawid.staudacher@uniklinik-freiburg.de (D.L.S.); 2 Department of Cardiology and Angiology, Heart Center Freiburg University, Faculty of Medicine, University of Freiburg, 79106 Freiburg, Germany; jonathan.rilinger@uniklinik-freiburg.de (J.R.); markus.jaeckel@uniklinik-freiburg.de (M.J.); 3 Department of Hematology, Oncology, and Stem Cell Transplantation, Faculty of Medicine, Freiburg University Medical Center, 79106 Freiburg, Germany; manuel.holst@uniklinik-freiburg.de; 4 Department of Nephrology, Faculty of Medicine, Freiburg University Medical Center, 79106 Freiburg, Germany; felix.rottmann@uniklinik-freiburg.de; 5 Department of Cardiovascular Surgery, Heart Center, Faculty of Medicine, University of Freiburg, 79106 Freiburg, Germany; christoph.benk@uniklinik-freiburg.de; 6 Heidelberg Institute of Global Health, University of Heidelberg, 69117 Heidelberg, Germany

**Keywords:** extracorporeal membrane oxygenation, veno-arterial ECMO, veno-venous ECMO, carbon monoxide, survival

## Abstract

Background: Patients supported with extracorporeal membrane oxygenation (ECMO) may develop elevated carboxyhemoglobin (CO-Hb), a finding described in the context of hemolysis. Clinical relevance of elevated CO-Hb in ECMO is unclear. We therefore investigated the prognostic relevance of CO-Hb during ECMO support. Methods: Data derives from a retrospective single-center registry study. All ECMO patients in a medical ICU from October 2010 through December 2019 were considered. Peak arterial CO-Hb value during ECMO support and median CO-Hb values determined by point-of-care testing for distinct time intervals were determined. Groups were divided by CO-Hb (<2% or ≥2%). The primary endpoint was hospital survival. Results: A total of 729 patients with 59,694 CO-Hb values met the inclusion criteria. Median age (IQR) was 59 (48–68) years, 221/729 (30.3%) were female, and 278/729 (38.1%) survived until hospital discharge. Initial ECMO configuration was veno-arterial in 431/729 (59.1%) patients and veno-venous in 298/729 (40.9%) patients. Markers for hemolysis (lactate dehydrogenase, bilirubin, hemolysis index, and haptoglobin) all correlated significantly with higher CO-Hb (*p* < 0.001, respectively). Hospital survival was significantly higher in patients with CO-Hb < 2% compared to CO-Hb ≥ 2%, evaluating time periods 24–48 h (48.6% vs. 35.2%, *p* = 0.003), 48–72 h (51.5% vs. 36.8%, *p* = 0.003), or >72 h (56.9% vs. 31.1%, *p* < 0.001) after ECMO cannulation. Peak CO-Hb was independently associated with lower hospital survival after adjustment for confounders. Conclusions: In ECMO, CO-Hb correlates with hemolysis and hospital survival. If high CO-Hb measured should trigger a therapeutic intervention in order to reduce hemolysis has to be investigated in prospective trials.

## 1. Background

Ambient air carbon monoxide (CO) originates from incomplete combustion [1,2]. CO has a very high affinity to hemoglobin by forming carboxyhemoglobin (CO-Hb) and thereby superseding oxygen (O_2_), reducing oxygen delivery by the blood. Higher concentrations may ultimately lead to tissue hypoxemia and serious organ damage [3]. In humans, blood CO concentrations do not necessarily correlate with toxicity [3]. Even comparably low CO concentrations may damage the heart and brain by affecting mitochondrial respiration and cellular energy utilization, leading to inflammatory response and formation of free radicals [3,4].

High CO-Hb values are frequently found in smokers and have been connected to many of the adverse events connected to smoking. This correlation is conflicted by lower in-hospital mortality in acute coronary syndromes of smokers compared to non-smokers; a phenomenon coined smoking paradox [5]. Importantly, ambient air and smoking are not the only sources of CO. For several diseases, such as chronic obstructive pulmonary disease or hemolytic anemia, increased blood CO concentrations have been described [6,7]. Endogenous CO on the one hand may derive from increased heme catabolism [6,7], on the other hand endogenous CO is increased as part of the electrophilic stress response [4]. Both hemolysis and stress are frequently observed in critically ill patients on the intensive-care unit (ICU) and particularly in patients supported with extracorporeal membrane oxygenation (ECMO) [8,9,10]. Apart from its toxic characteristics in higher concentrations, carbon monoxide is known to be involved in signal transductions, a mechanism which is referred to as gasotransmission [11].

Several reports describe elevated CO-Hb concentrations in patients on ECMO [12,13,14,15,16]. Increased levels of CO-Hb up to a certain degree are to be expected in the ECMO collective, since hemolysis is a common complication of extracorporeal circulation and as discussed above hemolysis is a source of endogenous CO. A recent review of hemolysis in VV ECMO reported an incidence of significant hemolysis in up to 29% of investigated patients [17]. This hemolysis is partly caused by the centrifugal pumps being an integral part of every ECMO [18]. However, inflammation caused by the ECMO tubing and shear stress might also contribute to hemolysis [19]. As for VA ECMO, data suggest even higher incidences of hemolysis in VA compared to VV ECMO [8]. Several reports associate hemolysis in ECMO with renal failure, thrombotic events, transfusions, and mortality [19]. It is unclear if hemolysis *per se* is responsible for adverse prognosis in ECMO or if hemolysis is just a marker of poor prognosis [19].

On the other hand, ECMO has been successfully deployed in CO poisoning [20] and animal data exists suggesting improved survival after extracorporeal cardiopulmonary resuscitation in pigs treated with carbon monoxide [21].

Taken together, the role of CO-Hb in patients on ECMO is unclear. Therefore, we conducted this single-center registry study to assess the prevalence of elevated CO-Hb in ECMO patients and to evaluate a potential correlation of CO-Hb and the primary endpoint of hospital survival.

## 2. Methods

Study setting: We conducted a single-center retrospective registry study. Data derives from a registry of all patients on ECMO, veno-arterial (VA ECMO) as well as veno-venous (VV ECMO), treated at a medical intensive care unit (ICU) located at a tertiary university hospital. Exclusion criteria from this registry were cannulation in the operation theatre and treatment at a different intensive care unit. All patients receiving ECMO support from October 2010 until December 2019 were included in this analysis. Patients received VA ECMO for the treatment of hypoperfusion, shock, or even cardiac arrest (extracorporeal cardiopulmonary resuscitation, ECPR), and VV ECMO in severe respiratory failure.

Local ECMO setting: Our center provides a 24/7 ECMO service. As for local policy, decision to cannulate for ECMO is made after multidisciplinary discussion at the bedside according to established criteria [22,23,24]. ECPR was defined as VA ECMO cannulation during continuous cardiopulmonary resuscitation without return of spontaneous circulation (ROSC); or as VA ECMO cannulation within the first 20 min after ROSC in case of persistent hemodynamic instability as previously suggested [25]. ECMO cannulation and operation were done according to regularly revised standard operating procedures as described earlier [26,27].

Blood gas analysis (BGA): BGA were processed as described earlier [27]. BGA test results were automatically transferred to the electronic patient files. Frequency of point of care testing (POCT) was driven by clinical judgement of the ICU staff. According to local standard procedures, in patients supported with ECMO at least every four hours a POCT BGA was performed as well as before and after cannulation. Missing samples are typically attributed to out-of-hospital cannulation, technical errors, or in case of missing patient identification of the blood sample. A minimum of 18 different values including CO-Hb were routinely measured with each blood gas analysis.

Data acquisition and statistical analysis: All data for this study derived from a single-center retrospective registry. Parameters from the last 24 h before ECMO and for the total duration of ECMO support were automatically extracted from the patient records and median values for CO-Hb were calculated for predefined time intervals (−24–0 h, 0–24 h, 24–48 h, 48–72 h, and 72 h till end of ECMO support) after ECMO initiation. For serum markers of hemolysis (free hemoglobin, LDH, bilirubin, haptoglobin, and hemolysis index), all measurements were extracted and the nearest CO-Hb value was determined. For correlation of CO-Hb with serum markers of hemolysis (LDH, bilirubin, haptoglobin, and hemolysis index), significance was calculated using 1-way ANOVA. For survival analysis, a predefined cut-off was used in order to divide the cohort into two groups, one with low peak CO-Hb (<2%) and one group with high peak CO-Hb (≥2%) as suggested previously [28].

We also recorded patient demographics and medical history, occurrence of important events (e.g., decannulation, death, and discharge from ICU). Multivariate logistic regression analysis was performed for hospital survival as the dependent variable and known predictors of survival in patients with ECMO (ECMO configuration, age, lactate, pH, female gender), comorbidities (coronary heart disease, liver-, renal-, lung disease, and diabetes mellitus) and blood gas analysis parameters (CO-Hb, paO_2_, paCO_2_, hemoglobin) as independent variables. Since markers of hemolysis taken from routine blood analyses were not available in proximity to POCT of the highest CO-Hb for many patients, these routine blood markers were not included in the logistic regression analysis.

For data analysis and visualization, SPSS (version 23, IBM Statistics, Armonk, NY, USA), Python programming language (version 3.8.3, Python Software Foundation, Wilmington, DE, USA), seaborn [29], and Prism (version 8, GraphPad, San Diego, CA, USA) were used. For statistical analysis, unpaired t-test, Fisher’s-exact/chi-square test, 1-way ANOVA, and Log-rank/Gehan Breslow test were used as applicable. Correlations were evaluated computing Pearson correlation coefficients. A *p*-value < 0.05 was considered statistically significant. All categorical variables are presented in absolute number (percent of all patients), while continuous variables are presented as median (interquartile range), if not stated otherwise.

## 3. Results

Patient cohort: 740 ECMO patients were identified. Of these, 11/740 (1.5%) patients had to be excluded since no point of care blood gases were documented. Therefore, a total of 729 patients were considered for this research (Supplementary Figure S1). Median age at the time of ECMO cannulation was 59 (48–68) years, and 221/729 (30.3%) patients were female. A total of 431/729 patients (59.1%) were supported with VA ECMO and 298/729 patients (40.9%) received VV ECMO.

Considering all patients, 278/729 (38.1%) survived until hospital discharge (Table 1). Survival to discharge was higher in patients supported with VV ECMO compared to patients on VA ECMO (145/431 (33.6%) vs. 133/298 (44.6%), *p* = 0.003, Table 1). Other parameters including sex, body measurements, and known comorbidities were similar between survivors and non-survivors, see Table 1.

**Figure 1:**
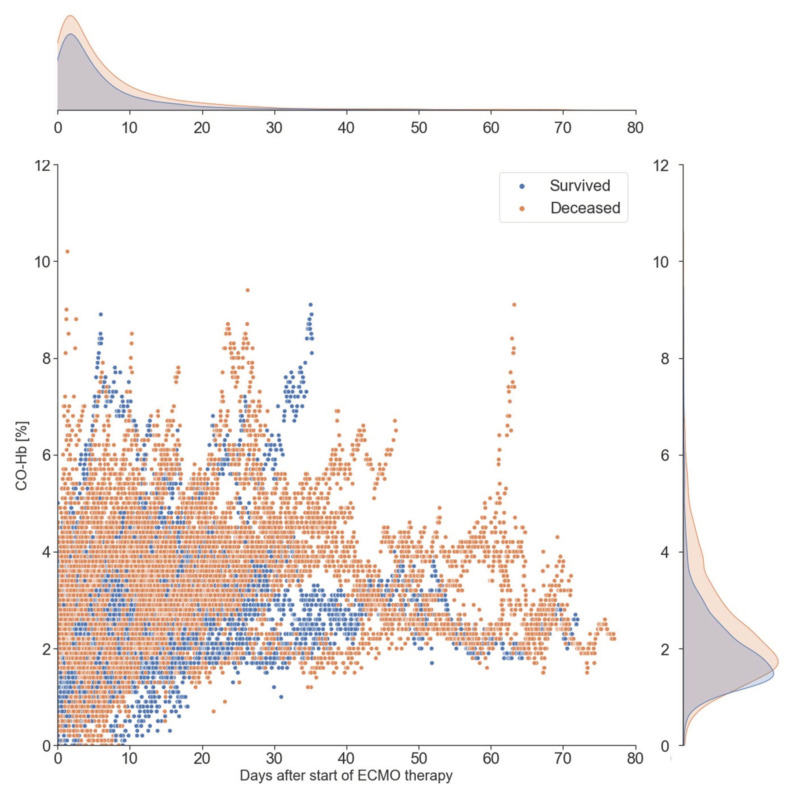
Progression of arterial CO-Hb during ECMO therapy. A total of 59,694 unique arterial blood gas analysis during ECMO support for 729 different patients were analyzed and plotted over time for CO-Hb. Here, we can see that mean CO-Hb rises the longer the ECMO support is ongoing. Abbreviations: *CO-Hb* carboxyhemoglobin, *ECMO* extracorporeal membrane oxygenation therapy.

Blood gas analysis results: In total, 59,694 independent arterial blood gas analyses (BGA) were identified. During the course of ECMO therapy, median arterial CO-Hb increased from 1.5% (IQR 1.2–1.8%) in samples taken during the first day of ECMO support to 1.9% (1.5–2.4%) in samples taken from patients with ECMO support >72 h (*p* < 0.001). This steady increase in CO-Hb over the duration of ECMO is illustrated in Figure 1.

**Figure 2:**
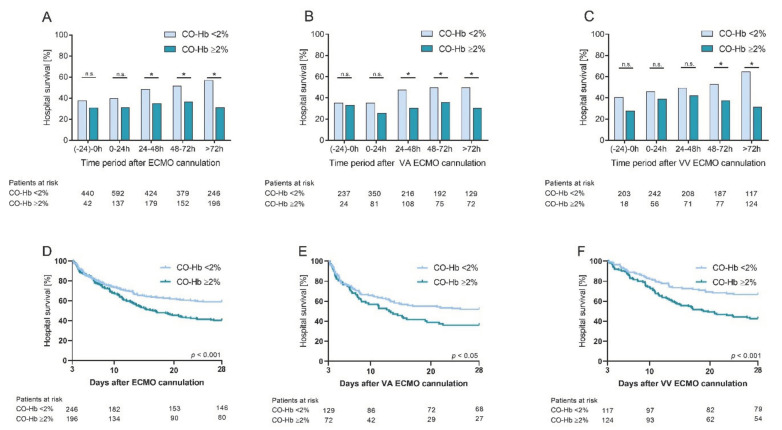
Survival in relation to CO-Hb levels. (**A**) Survival in relation to median CO-Hb under or over 2% at different time intervals prior and during ECMO support. Survival is significantly lower in the high CO-Hb group for 24–48 h, 48–72 h, and >72 h (*p* = 0.003, 0.003, and <0.001, respectively). (**D**) Kaplan–Meier survival curve for median CO-Hb >72 h after cannulation, separated in high and low CO-Hb-group (*p* < 0.001). (**B**,**E**) shows subgroup analysis for VA ECMO patients whereas for (**C**,**F**) only VV ECMO patients where included. Abbreviations: * significant, *CO-Hb* carboxyhemoglobin, *ECMO* extracorporeal membrane oxygenation therapy, *n.s.* not significant, *VA* veno-arterial, *VV* veno-venous.

CO-Hb and hospital survival: Comparing patients with median CO-Hb < 2% to patients with median CO-Hb ≥ 2% and only considering samples taken prior to ECMO cannulation or the first 24 h after cannulation, survival was not significantly different between the groups (Figure 2A). Considering samples taken from patients with ECMO support above 24 h, patients with a median CO-Hb < 2% had a statistically better hospital survival compared to patients with CO-Hb ≥ 2%. This finding was confirmed in Kaplan–Meier survival curves (Figure 2D). The better hospital survival in patients with CO-Hb < 2% was evident in both VA and VV ECMO (Figure 2B,C,E,F). In a multivariate logistic regression analysis including known predictors of hospital survival in ECMO as well as the BGA with peak CO-Hb for each individual patient, CO-Hb was an independent predictor of in-hospital mortality (*p* < 0.001). Other significant independent predictors of survival included age, lactate, pH, and hemoglobin; see Table 2.

**Figure 3:**
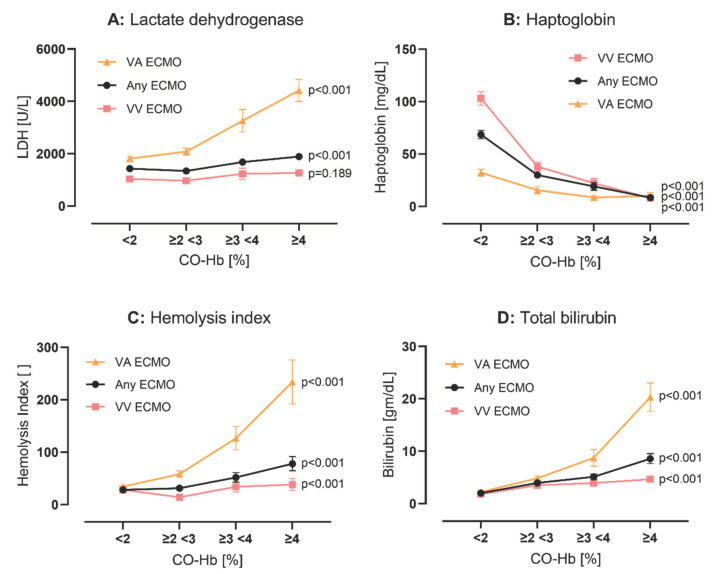
Hemolysis parameter for nearest CO-Hb. Blood marker of hemolysis ((**A**): LDH, (**B**): haptoglobin, (**C**): hemolysis index, and (**D**): bilirubin) are grouped into four groups according to nearest CO-Hb. Data are given as mean with standard error of mean and significance is calculated with ANOVA. Abbreviations: *CO-Hb* Carboxyhemoglobin, *LDH* lactate dehydrogenase, *VV ECMO* veno-venous extracorporeal membrane oxygenation, *VA ECMO* veno-arterial extracorporeal membrane oxygenation.

Hemolysis: Each marker of hemolysis during ECMO available in the dataset (total bilirubin n = 2132, hemolysis index n = 3540, haptoglobin n = 1015, and LDH n = 3507) was linked to the nearest CO-Hb as described above. For free hemoglobin, only 54 independent measurements were found for all patients. In groups of escalating Co-Hb, markers of hemolysis worsened significantly (*p* < 0.001 for each parameter, respectively, except for LDH in the VV ECMO subgroup, where *p* = 0.189; see Figure 3). All four markers of hemolysis correlation significantly with the respective Co-Hb values in all ECMO modes; see Supplementary Table S1.

## 4. Discussion

In this retrospective registry study of patients supported with ECMO, higher carboxyhemoglobin levels correlated with hemolysis and ultimately with lower hospital survival.

There are only a few reports describing the relationship between arterial CO-Hb and survival of ICU patients. Some previous reports described a positive correlation of CO-Hb and hospital mortality in non-ECMO ICU patients [30], while others suggested a positive correlation between arterial CO-Hb and survival [31]. In ECMO patients, data from case series suggested a correlation between elevated CO-Hb concentrations and death [14,15,32] but collectives have never been as large as in the present study. Our data also confirms that the correlation between CO-Hb and hospital survival described in ECMO patients indeed can be seen in both veno-arterial and veno-venous configuration. There are distinct differences between the two ECMO configurations. First: survival is higher in VV ECMO, a finding which fits nicely into literature and the ELSO registry. Second, blood markers of hemolysis correlate stronger with CO-Hb in the VA compared to the VV group. This finding has not yet been described in literature. A smaller case series observed the correlation on both ECMO configurations. We can only speculate on the reason for the weaker correlation in the VV ECMO group. Since baseline levels for LDH and haptoglobin however were different between both ECMO configurations, patient specific reasons might be responsible.

The underlying source of CO-Hb in this specific collective is difficult to assess. Typical sources like fire, engine exhaust, and continued smoking can be excluded in these patients [3]. Half-life values for CO-Hb under different conditions are 320 min (room air), 74 min (normobaric oxygen) and 20 min (100% hyperbaric oxygen) [3]. Patients cease to smoke while under ECMO support so it is safe to assume that after a day of therapy smoking related CO-Hb should be washed out from the patient. Therefore, the observed effects are most likely not due to prior smoking habit. For patients on ECMO support, a recent study did suggest increasing CO-Hb values as an early sign of oxygenator dysfunction, one cause of hemolysis in ECMO patients due to microthrombi [33]. Hemolysis is one the major contributors of endogenous CO-Hb as it is known that endogenous CO is produced by heme oxygenase 1 (HO-1) which is induced by oxidative stress, hypoxia, or heme derivates [34,35]. It is assumed that this process generates up to 85% of endogenous CO [31]. HO is essential in the conversion of heme to CO, iron, and biliverdin, a process especially important in hemolysis as increased amounts of heme have to be degraded [31]. An important question is why elevated CO-Hb levels impacts survival. The degradation of heme by HO is assumed to play a major role in human physiology and pathogenesis of a multitude of different diseases like diabetes, inflammation, heart disease, and pulmonary disease [36]. Thus, elevated CO-Hb could be a surrogate of disease severity. Literature however is not clear since some studies on ICU patients reported a negative correlation of CO-Hb and prognosis [37,38]. Also, no correlation of CO-Hb levels and disease severity was found in patients with liver cirrhosis [39]. Therefore, disease severity alone cannot be the only driver for CO-Hb and discriminating the effects of CO-Hb alone on mortality with the effects of the underlying disease is near to impossible.

When evaluating CO, smoking habits have to be taken into account since smoking increases CO-Hb [40]. On the one hand, CO-Hb above 5% and adverse prognosis correlate well in smokers [40]. On the other hand, lower in-hospital mortality in acute coronary syndromes has been reported in smokers [5]. This so called ‘smokers’ paradox’ can be detected in our data as well, with better short-term survival in smokers in the whole cohort and after adjustment for confounders. Since experimental data on CO could not demonstrate favorable hemodynamics, it is not clear if this correlation of smoking and prognosis is originated in potentially higher CO in smokers [41]. As arterial CO-Hb levels are lowest early after ECMO cannulation, smoking status might be a minor bias to this registry.

In the ECMO collective specifically, CO-Hb might be elevated due to hemolysis [12,15,16]. Hemolysis derived CO-Hb was especially discussed in the context of CO-Hb levels above 7% [13,15]. In our study, we see all four markers or hemolysis investigated escalating alongside CO-Hb. This is a strong indicator for hemolysis being a dominant driver of CO-Hb generation. At the bedside, hemolysis should be suspected and validated by measuring the standard hemolysis parameters in the case of elevated or rising CO-Hb values in point of care testing for patients on ECMO support.

## 5. Limitations

Based on the retrospective, observational, single-center design, limitations and biases have to be considered and our findings should be considered hypothesis generating only. Data was collected retrospectively and therefore incomplete documentation, for example on the smoking status, is a limiting factor. This is a significant potential bias, as the anamnesis of concomitant disease like cardiovascular risk factors might be incomplete in non-survivors, especially those who died early. Particularly the interpretation of smoking status is limited, since no data on individual baseline CO-Hb prior to ECMO support was available. Concerning hemolysis, plasma-free hemoglobin is not routinely measured at our center and therefore cannot be used as gold standard for hemolysis in this registry.

## 6. Conclusions

In this retrospective analysis, CO-Hb was an independent predictor of survival in patients supported with veno-arterial and veno-venous ECMO. Whereas elevated CO-Hb values are exclusively caused by hemolysis, remains uncertain. Future research, especially from prospective studies, is required to determine if CO-Hb should be considered a therapeutic target in patients on extracorporeal membrane oxygenation.

## Figures and Tables

**Table 1:** Patient characteristics and path of ICU stay.

	Deceased (451 Patients)	Survived (278 Patients)	All Patients (729 Patients)	*p*-Value
Age [years]	61 (51–70)	55.5 (45–65)	59 (48–68)	<0.001
Female	136 (30.16%)	85 (30.58%)	221 (30.32%)	0.905
BMI [kg/m^2^]	26.37 (24.15–27.76)	24.89 (23.44–29.32)	25.95 (23.92–28.01)	0.272
CHD	176 (39.02%)	89 (32.01%)	265 (36.35%)	0.056
Hypertension	167 (37.03%)	115 (41.37%)	282 (38.68%)	0.243
Liver disease	36 (7.98%)	14 (5.04%)	50 (6.86%)	0.126
Renal disease	59 (13.08%)	31 (11.15%)	90 (12.35%)	0.441
Diabetes	95 (21.06%)	54 (19.42%)	149 (20.44%)	0.594
Lung disease	96 (21.29%)	59 (21.22%)	155 (21.26%)	0.984
Active Smoker ^a^	108 (25.4%)	99 (36.5%)	207 (29.7%)	0.001
ECMO duration [days]	3.45 (1–7.36)	4.85 (2.99–7.82)	4.05 (1.99–7.59)	0.119
Duration of ICU stay [days]	3.93 (1.05–10.56)	28 (28–28.43)	13.14 (2.53–28)	<0.001
ECMO-setting				0.003
VA ECMO	286 (63.41%)	145 (52.16%)	431 (59.12%)	
VV ECMO	165 (36.59%)	133 (47.84%)	298 (40.88%)	

Patient characteristics of patients included in the present study. ^a^ smoking status not available for 33 patients. Abbreviations: *BMI* body mass index, *CAD* coronary artery disease, *ICU* intensive care unit, *VA ECMO* veno-arterial extracorporeal membrane oxygenation, *VV ECMO* veno-venous extracorporeal membrane oxygenation.

**Table 2:** Univariate and multivariate logistic regression analysis for hospital survival.

	Univariate Logistic Regression			Multivariate Logistic Regression		
	OR	(95% CI)	*p*-Value	OR	(95% CI)	*p*-Value
BMI [kg/m^2^]	1.02	(0.99–1.04)	0.234			
Female gender	1.05	(0.76–1.45)	0.779			
Age [years]	0.98	(0.97–0.99)	0.001	0.97	(0.96–0.99)	0.001
CAD	0.73	(0.53–1.00)	0.049	1.02	(0.68–1.54)	0.912
Liver disease	0.62	(0.33–1.16)	0.136			
Renal disease	0.82	(0.52–1.31)	0.413			
Diabetes mellitus	0.90	(0.62–1.31)	0.577			
Lung disease	0.99	(0.69–1.43)	0.965			
Active smoker	1.66	(1.20–2.31)	0.002	1.58	(1.08–2.30)	0.019
CO-Hb (peak)	0.87	(0.77–0.98)	0.023	0.72	(0.61–0.84)	0.001
Lactate [mmol/l] ^a^	0.80	(0.76–0.85)	0.001	0.81	(0.76–0.86)	0.001
pH ^a^	644.96	(105–3959)	0.001	9.35	(0.97–89.89)	0.053
paCO_2_ [mmHg] ^a^	1.00	(0.99–1.02)	0.721			
paO_2_ [mmHg] ^a^	1.00	(0.99–1.00)	0.001	1.00	(1.00–1.00)	0.772
Hb [g/dl] ^a^	1.23	(1.12–1.35)	0.001	1.15	(1.02–1.29)	0.021

Predictors of hospital survival. ^a^ at time point of peak CO-Hb. Abbreviations: *BMI* body mass index, *CAD* coronary artery disease, *CI* confidence interval, *CO-Hb* carboxyhemoglobin, *Hb* hemoglobin, *OR* odds ratio, *paCO_2_* partial arterial pressure of carbon dioxide, *paO_2_* partial arterial pressure of oxygen.

## Data Availability

The datasets used and analyzed during the current study are available from the corresponding author on reasonable request.

## Supplementary Materials

The following supporting information can be downloaded at: https://www.mdpi.com/article/10.3390/diagnostics12071642/s1, Figure S1: patient selection; Table S1: Correlation of hemolysis parameter for nearest CO-Hb.

## Abbreviations

BMI	Body mass index
CAD	Coronary artery disease
CO	Carbon monoxide
CO-Hb	Carboxyhemoglobin
ECPR	Extracorporeal cardiopulmonary resuscitation
Hb	Hemoglobin
ICU	Intensive care unit
IHCA	In hospital cardiac arrest
LDH	Lactate dehydrogenase
OHCA	Out of hospital cardiac arrest
pCO_2_	Partial pressure of carbon dioxide
pO_2_	Partial pressure of oxygen
POCT	Point of care testing
ROSC	Return of spontaneous circulation
VA ECMO	Veno-arterial extracorporeal membrane oxygenation
VV ECMO	Veno-venous extracorporeal membrane oxygenation
