# Supplementary material for: Carboxyhemoglobin (CO-Hb) Correlates with Hemolysis and Hospital Mortality in Extracorporeal Membrane Oxygenation: A Retrospective Registry
Source: Diagnostics (Basel). 2022 Jul 5;12(7):1642. doi: 10.3390/diagnostics12071642 (PMC9324470; doi:10.3390/diagnostics12071642)
Supplement: Supplementary file 1 [file diagnostics-12-01642-s001.zip › diagnostics-1733059-supplementary.pdf]

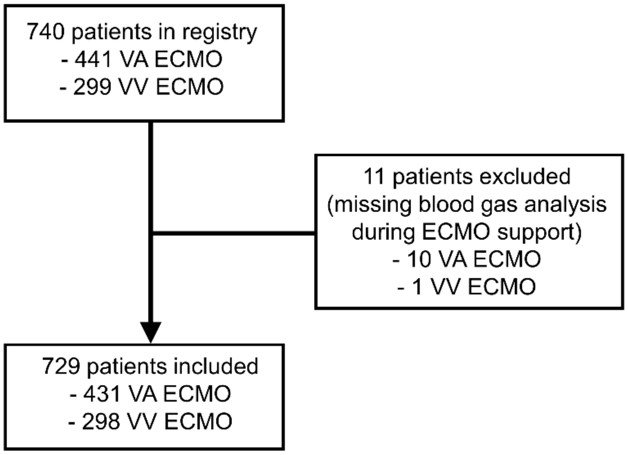

Figure S1. Patient selection.

Table S1. Correlation of hemolysis parameter for nearest CO-Hb.

	**Any ECMO**		**VA ECMO**		**VV ECMO**	
	r^2	*p*-value	r^2	*p*-value	r^2	*p*-value
LDH	0.0027	<0.0001	0.0341	<0.0001	0.0023	0.0276
Haptoglobin	0.0648	<0.0001	0.0401	<0.0001	0.1343	<0.0001
Hemolysis index	0.0138	<0.0001	0.0692	<0.0001	0.0094	<0.0001
Bilirubin	0.0014	<0.0001	0.2117	<0.0001	0.0002	0.0188

Blood markers of hemolysis were correlated to the nearest CO-Hb available using Pearson's correlation. Table gives r^2 and significance for each of the four markers of hemolysis (LDH, haptoglobin, hemolysis index, and bilirubin) investigated. Abbreviations: *LDH* lactate dehydrogenase, *VV ECMO* veno-venous extracorporeal membrane oxygenation, *VA ECMO* veno-arterial extracorporeal membrane oxygenation.
